# Comparison of remimazolam tosylate and sevoflurane for anesthesia induction with preserved spontaneous respiration during tracheal intubation: a prospective, single-center, randomized controlled trial

**DOI:** 10.1186/s13741-025-00588-8

**Published:** 2025-10-01

**Authors:** Yu Hong, Shiyu Meng, Jiayi Liu, Qiong Zhao, Jun Peng, Yuqing Chen

**Affiliations:** 1 Department of Anesthesiology, Sun Yat-Sen Memorial Hospital of Sun Yat-Sen University, Guangzhou, China; 2 Department of Anesthesiology, The First People’s Hospital of Kashi Prefecture, Affiliated Kashi Hospital of Sun Yat-Sen University, Kashi, China

**Keywords:** Remimazolam, Sevoflurane, Spontaneous breathing, Anesthesia induction, Endotracheal intubation, Safety, Efficacy

## Abstract

**Background and objectives:**

Remimazolam tosylate is a novel anesthetic agent known for its rapid onset, non-irritating and non-polluting properties, effective sedation with minimal respiratory depression, short duration of action, and suitability for continuous infusion. Additionally, it can be efficiently antagonized by flumazenil. This study aims to explore the feasibility and safety of Remimazolam tosylate for anesthesia induction while maintaining spontaneous respiration.

**Methods:**

This prospective, randomized controlled trial involved patients aged 18–65 years with non-difficult airways who were scheduled for endotracheal intubation under general anesthesia. Participants, after receiving the same protocol of dexmedetomidine sedation and Lidocaine surface anesthesia, were randomly assigned to either the sevoflurane induction group or the remimazolam tosylate induction group, with 30 patients in each group. Anesthesia induction was performed while maintaining spontaneous respiration, followed by endotracheal intubation.

**Results:**

All enrolled patients successfully underwent intubation, and no severe respiratory depression or other complications were observed in either group (Successful anesthesia induction intubation is defined as follows: (1) successful anesthesia induction without the need for rescue measures during the induction of anesthesia, (2) no awakening during anesthesia induction, (3) spontaneous breathing is preserved throughout the entire procedure, and (4) successful completion of intubation.). There were no statistically significant differences in heart rate, blood pressure, oxygen saturation, or blood gas results between the groups. Regarding the time required for anesthesia induction, the average time to achieve the condition for topical anesthesia to the throat was 7.3 min in the remimazolam group and 17.7 min in the sevoflurane group, which was statistically significant (*P* < 0.01). The total time to complete intubation was 11.4 min in the remimazolam group and 21.3 min in the sevoflurane group, which was statistically significant (*P* < 0.01). During the local anesthetic throat spray procedure, 47% of patients in the sevoflurane group and 80% in the remimazolam group experienced coughing. This difference was statistically significant. During intubation, 20% of patients in the sevoflurane group and 33% in the remimazolam group continued to cough, but this difference was not statistically significant..

**Conclusion:**

Compared with sevoflurane, remimazolam can be safely and effectively used for intubation while preserving spontaneous breathing, with a shorter time to achieve conditions for topical anesthesia and intubation.

## Background

Endotracheal intubation is a high-risk procedure in clinical anesthesia practice. Since many patients undergo endotracheal intubation only after anesthesia induction, when consciousness is lost and respiration ceases, the risk of asphyxiation or even death is imminent if oxygenation fails (Ahmad et al. 2020; Aziz and Kristensen 2020). Maintaining spontaneous respiration during anesthesia induction and intubation could significantly improve patient safety (Ahmad et al. 2019; Zhou et al. 2019; Wang et al. 2018). However, this approach, which aims to preserve spontaneous respiration while ensuring both safety and comfort, is challenging to implement clinically. The difficulty arises from the fact that traditional intravenous anesthetics cannot simultaneously provide sufficient depth of anesthesia to suppress the stimulation from intubation while also preserving spontaneous respiration and ensuring oxygenation (Wang et al. 2021; Johnson 2012).

Currently, an anesthesia protocol centered on the inhalation anesthetic sevoflurane is often used for maintaining spontaneous respiration during anesthesia induction and intubation (Aziz and Kristensen 2020). However, this protocol has significant drawbacks. Sevoflurane has an unpleasant odor, poses a risk of air pollution, and requires a tightly sealed face mask, which can be uncomfortable for the patient. Moreover, achieving the required depth of anesthesia for intubation demands prolonged inhalation of sevoflurane (three time constants: over 10 min), which can increase the risk of anesthesia, particularly the risk of respiratory failure (Sneyd 2012).

Previous clinical trials have demonstrated that the novel intravenous anesthetic remimazolam tosylate offers several advantages: rapid onset, no irritation or pollution, effective sedation with minimal respiratory depression, short duration of action, suitability for continuous infusion, and efficient antagonism by flumazenil (Sneyd 2012; Wesolowski et al. 2016; Hu et al. 2022). However, it remains unclear whether remimazolam tosylate can be used for anesthesia induction to rapidly provide sufficient depth of anesthesia to suppress intubation stimuli while preserving spontaneous respiration, ensuring oxygenation, and enhancing safety.

Therefore, this study aims to explore the safety and efficacy of remimazolam tosylate for maintaining spontaneous respiration during anesthesia induction and endotracheal intubation. The goal is to offer new insights and methods for anesthesia induction with spontaneous respiration, provide more options for patient comfort, and contribute to the safe management of unanticipated difficult airways or known difficult airways.

## Materials and methods

### Study design

The present prospective, single-center, single-blind, randomized, controlled trial was carried out between January 2023 and October 2023 in Sun Yat-Sen Memorial Hospital of Sun Yat-Sen University. Our protocol gained approval from the Ethical Committee of Sun Yat-Sen Memorial Hospital, Sun Yat-Sen University (Guangzhou, China) (No. 2020-KY-46). Every patient or the legal representative provided informed consent prior to performing these procedures. This study was performed in accordance with the Consolidated Standards of Reporting Trials (CONSORT) criteria and the Declaration of Helsinki, registered in the Chinese Clinical Trial Registry (https://www.chictr.org.cn, No. ChiCTR2100048242).

### Patient inclusion and exclusion criteria

We plan to enroll 60 patients aged 18–65 who undergo general anesthesia with tracheal intubation, and the patient will be ruled out of the trial when presenting with below conditions: (1) weight less than 40 kg or BMI > 30 kg/m^2^; (2) have the following diseases: central nervous system diseases, hypertension (grade 2 or above) (McEvoy et al. 2024), heart disease (NYHA grade 2 or above) (Caraballo et al. 2019); (3) preoperatively predicted difficult airway, history of tracheotomy, upper respiratory obstruction, obstructive sleep apnea–hypopnea syndrome, nasopharyngeal cavity abnormalities, and other diseases affecting normal ventilation; (4) lung diseases affecting normal oxygenation (pneumonia, chronic obstructive pulmonary disease, asthma, bronchiectasis, emphysema, silicosis, pneumoconiosis, post-lobectomy, etc.); and (5) diseases predisposing to regurgitation and aspiration during anesthetic induction.

### Randomization and blinding

These patients will be randomly allocated in a 1:1 ratio to two groups—the sevoflurane induction group (SF group, *n* = 30, control) and the remimazolam tosylate induction group (RZ group, *n* = 30, experimental)—using a computer-generated random number table. Allocation concealment will be ensured via sequentially numbered, opaque, sealed envelopes in this single-blinded trial with participants blinded.​

### Procedures

**Fig. 1:**
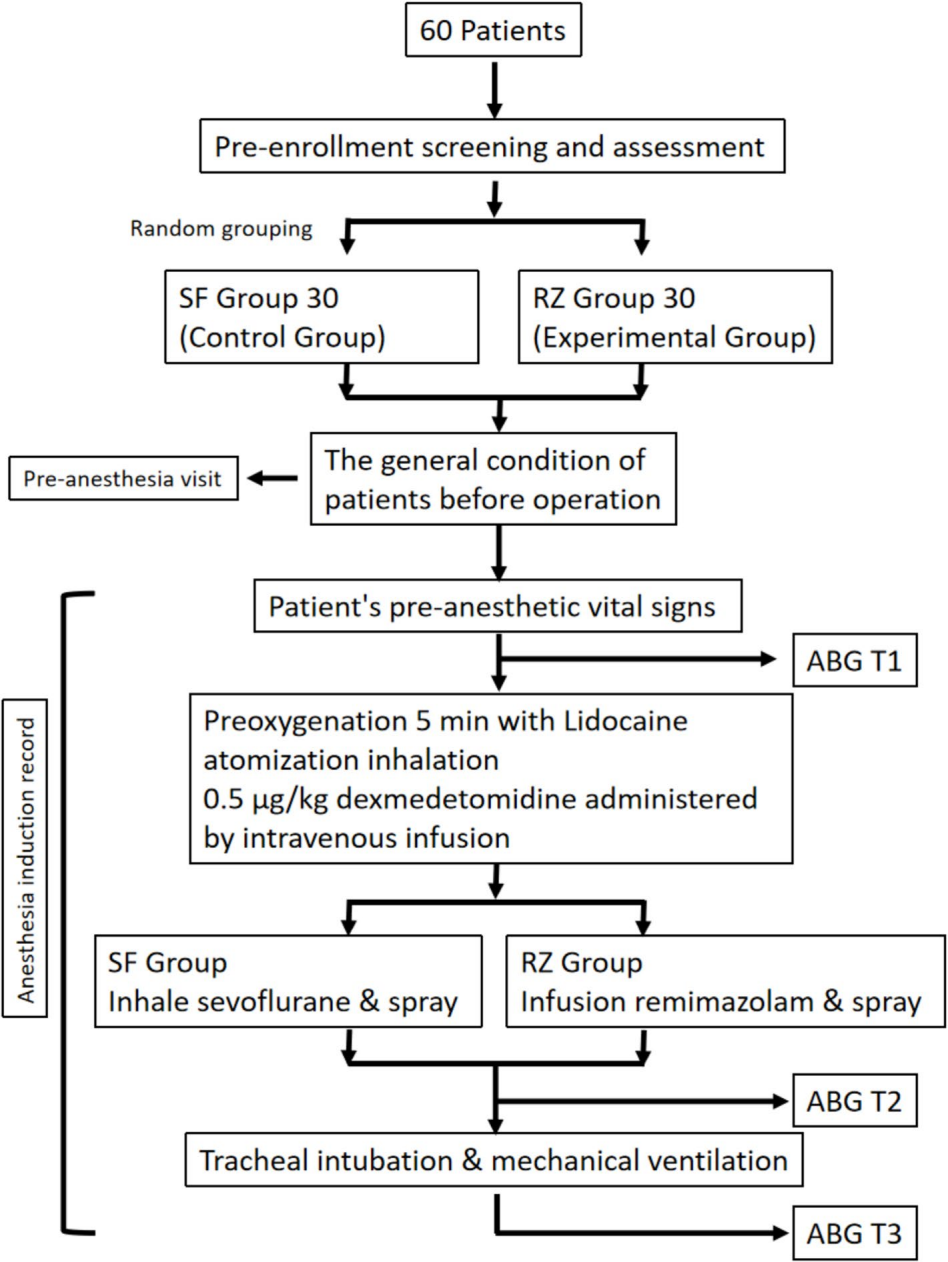
Research roadmap. SF, sevoflurane, RZ, remimazolam, ABG, arterial blood gas analysis

No premedication will be administered to any patient. Upon entering the operating room, routine monitoring and Narcotrend EEG monitoring will be established. To ensure the safety of anesthesia induction and accurate blood pressure monitoring during the trial, as well as to facilitate arterial blood gas analysis for comparing the patient’s oxygenation index and blood lactate levels, all patients underwent arterial catheterization for blood pressure monitoring. This also aided in intraoperative hemodynamic management. The procedure will be carried out as follows (Fig. 1):

- *Step 1:* The patients will breathe ambient air while radial arterial catheterization is performed for continuous blood pressure monitoring, followed by the first arterial blood gas analysis.
- *Step 2:* Both groups will receive 6 L/min of 100% oxygen for 5 min via a face mask that fits tightly over the face. During this period, 10 mL of 2% lidocaine will be administered via nebulization (NE-C99, OMRON, China). Simultaneously, 0.5 µg/kg dexmedetomidine diluted in 100 mL of normal saline will be infused intravenously.
- *Step 3:* Induction procedures for the two groups will differ as follows:
- *SF group (control group):* Sevoflurane (Kaiteli, Shanghai Hengrui) will be used for induction, with an oxygen flow rate of 5 L/min. The concentration of sevoflurane will be increased incrementally, starting at 0.5% and increasing by 0.5% every three breaths until it reaches 5%, which will be maintained for 5 min. Once the patient’s eyelash reflex disappears, heart rate and breathing slow and stabilize, and no physical response occurs to chin lift, a video laryngoscope (UE, Zhejiang UE Medical) will be used to assess airway conditions. After spraying 3 mL of 2% lidocaine (lidocaine hydrochloride injection, Shanxi Shuanghe) onto the throat by a single-use drug delivery nebulizer (Aolishu AMW150-30, Nanchang Aomei Medical), the mask will be reapplied, and sevoflurane inhalation will continue for another minute before performing video laryngoscope-assisted intubation and a second arterial blood gas analysis.
- *RZ group (experimental group):* Remimazolam tosylate (Rebenz, Jiangsu Hengrui) will be administered intravenously at a concentration of 1 mg/mL and a rate of 6 mg/kg/h. After 3 min, the infusion rate will be adjusted to 1 mg/kg/h. The airway will then be assessed using a video laryngoscope, and 3 mL of 2% lidocaine will be sprayed onto the throat by a single-use drug delivery nebulizer (Aolishu AMW150-30, Nanchang Aomei Medical). After reapplying the mask and administering oxygen for 1 min, video laryngoscope-assisted intubation will be performed, followed by a second arterial blood gas analysis.
- *Step 4:* After successful intubation, both groups will receive an intravenous injection of 10 mg cisatracurium besylate (Jiangsu Hengrui) and 10 µg sufentanil (Yichang Renfu). Mechanical ventilation will be initiated (VT 8 mL/kg, RR 12 breaths/min, I:E 1:2), and sevoflurane inhalation will continue at 3% with an oxygen flow rate of 2 L/min. Immediately upon confirmation of successful intubation (based on the appearance of a capnography waveform after connecting to the ventilator and applying positive pressure), a third arterial blood gas analysis will be performed.

The three arterial blood gas analysis time points will be labeled as T1, T2, and T3 in chronological order. The following parameters will be recorded at each time point: blood gas values, heart rate, blood pressure, pulse oximetry (SpO_2_), and Modified Observer’s Assessment of Alertness/Sedation (MOAA/S) score (Chernik et al. 1990). All effects of induction and intubation will be systematically recorded and analyzed in the study, including respiratory depression, induction-related body movement, glottis exposure status, body movement during laryngoscopy, cough during topical anesthesia spray, cough during intubation, first-attempt intubation success rate, and procedure-related complications.

### Primary and secondary outcomes

#### Primary outcomes: success rate of intubation under anesthesia induction with preserved spontaneous breathing

Successful anesthesia induction intubation is defined as follows: (1) successful anesthesia induction without the need for rescue measures during the induction of anesthesia, (2) no awakening during anesthesia induction, (3) spontaneous breathing is preserved throughout the entire procedure, and (4) successful completion of intubation.

Secondary outcomes:

1. Anesthesia sedation time
  The anesthesia sedation time is defined as the period from the start of anesthesia induction (initiation of oxygen administration) until the patient achieves complete loss of consciousness. Specifically, it is the time from the start of oxygen administration until the patient reaches an MOAA/S score of 0. (This coincides with the initiation of local anesthetic throat spray.)
2. Anesthesia induction time
  The anesthesia induction time is defined as the period from the start of anesthesia induction (initiation of oxygen administration) until successful endotracheal intubation.

### Sample size and statistical analysis

In this study, sevoflurane was used as the control, and the success rate of intubation under anesthesia induction with preserved spontaneous breathing between the two groups was assumed to be 90%. *α* = 0.05 stood for significance, and the degree of assurance was set at 1-β = 80%. The sample size of *n* = 27 persons per group was calculated using SAS 9.4, and the shedding rate of each group was considered to be 10%. It was calculated that 30 people per group would be enrolled for analysis, and altogether 60 people would participate in this study.

SPSS software (Version 19.0, IBM Corp, Armonk, NY, USA) was employed for statistical analysis. Continuous data conforming to normal distribution were represented by mean ± standard deviation and examined by *t*-test. Homogeneity of variance was examined by a single-factor analysis of variance (ANOVA), whereas heterogeneity of variance was analyzed through a nonparametric test (Wilcoxon rank sum test). Differences between groups were compared by independent sample *t*-tests, whereas those in individual groups were compared by repeated measurement univariate analysis of variance. Categorical data were represented by percentages (%) and analyzed by a chi-square test or Fisher’s exact test. Rank data were analyzed by the rank sum test. *P* < 0.05 stood for statistical significance.

## Results

**Fig. 2:**
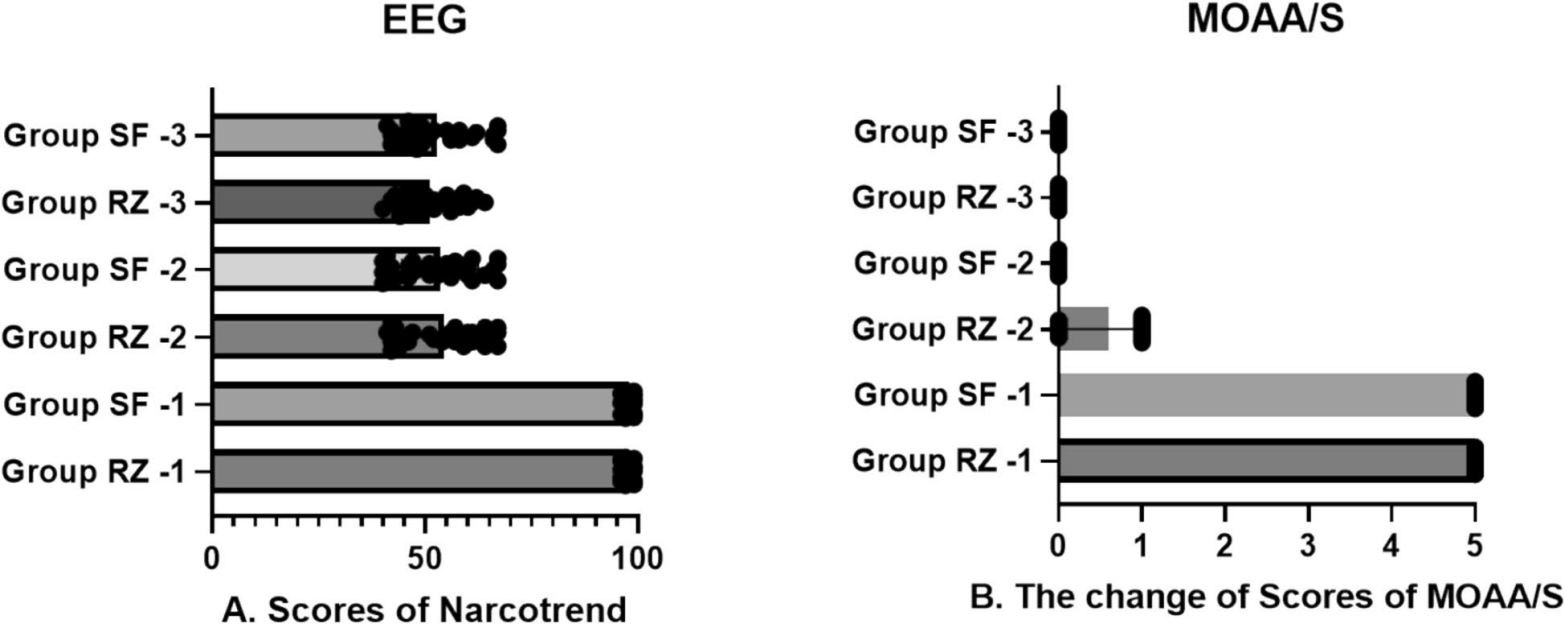
Comparison of anesthesia depth between the two groups at three time points. **A** There was no statistically significant difference in the EEG by Narcotrend between the two groups. **B** There was no statistically significant difference in MOAA/S between the two groups. EEG, electroencephalogram. MOAA/S modified observer’s assessment of alert/sedation scale. *P* < 0.05 was statistically significant

The baseline characteristics of the patients are presented in Table 1. The study included 30 patients in the sevoflurane group and 30 in the remimazolam group. Both groups achieved sufficient anesthetic depth during the study, as shown in Fig. 2. All enrolled patients successfully underwent intubation, and no severe respiratory depression occurred in either group.

**Table 1:** Summary of patient characteristics

Characteristic	Group (SF)	Group (RZ)	*t*/*χ*^2^ value	*P* value
Sex, *n* (%)			0.067	0.796
Male	15 (50)	14 (46.7)		
Female	15 (50)	16 (53.3)		
Age (year), mean ± SD	41.8 ± 10.5	47.1 ± 14.0	1.666	0.101
Height (cm), mean ± SD	161.3 ± 7.4	163.2 ± 6.5	1.058	0.294
Weight (kg), mean ± SD	64.1 ± 8.9	64.9 ± 8.4	0.388	0.700
BMI (kg/m^2^), mean ± SD	24.5 ± 2.0	24.3 ± 2.2	0.396	0.693

Data are represented by frequencies or means ± SD; SD, standard deviation; BMI, body mass index

No body movement was observed in the remimazolam group during induction, whereas 37% of patients in the sevoflurane group exhibited body movement during the excitation phase of anesthesia. Both groups had successful glottic exposure, with 10% of patients in the sevoflurane group and 20% in the remimazolam group showing body movement during the glottic exposure procedure, though the difference was not statistically significant. During the local anesthetic throat spray procedure, 47% of patients in the sevoflurane group and 80% in the remimazolam group experienced coughing, which was statistically significant. During intubation, 20% of patients in the sevoflurane group and 33% in the remimazolam group continued to cough, but this difference was not statistically significant.

No severe complications were observed in either group, including laryngospasm, bronchospasm, arrhythmias, myocardial ischemia, myocardial infarction, pulmonary edema, or cerebrovascular accidents (Table 2).

**Table 2:** The effect of induction and intubation

Events	Group (SF) *n* = 30 (%)	Group (RZ) *n* = 30 (%)	*t*/*χ*^2^ value	*P* value	RR
Respiratory depression	0 (0)	0 (0)	NA	NA	NA
Induction body movement	11 (37)	0 (0)	13.47	0.000*	0
Exposure of the glottis	30 (100)	30 (100)	NA	NA	NA
Body movement during exposure	3 (10)	6 (20)	1.176	0.278	2
Cough during spray	14 (47)	24 (80)	7.177	0.007*	1.7
Cough during intubation	6 (20)	10 (33)	1.364	0.243	1.65
Successful intubation	30 (100)	30 (100)	NA	NA	NA
Complication	0 (0)	0 (0)	NA	NA	NA

Data are represented by frequencies. *P < 0.01. *P* < 0.05 was statistically significant. RR, relative risk

**Fig. 3:**
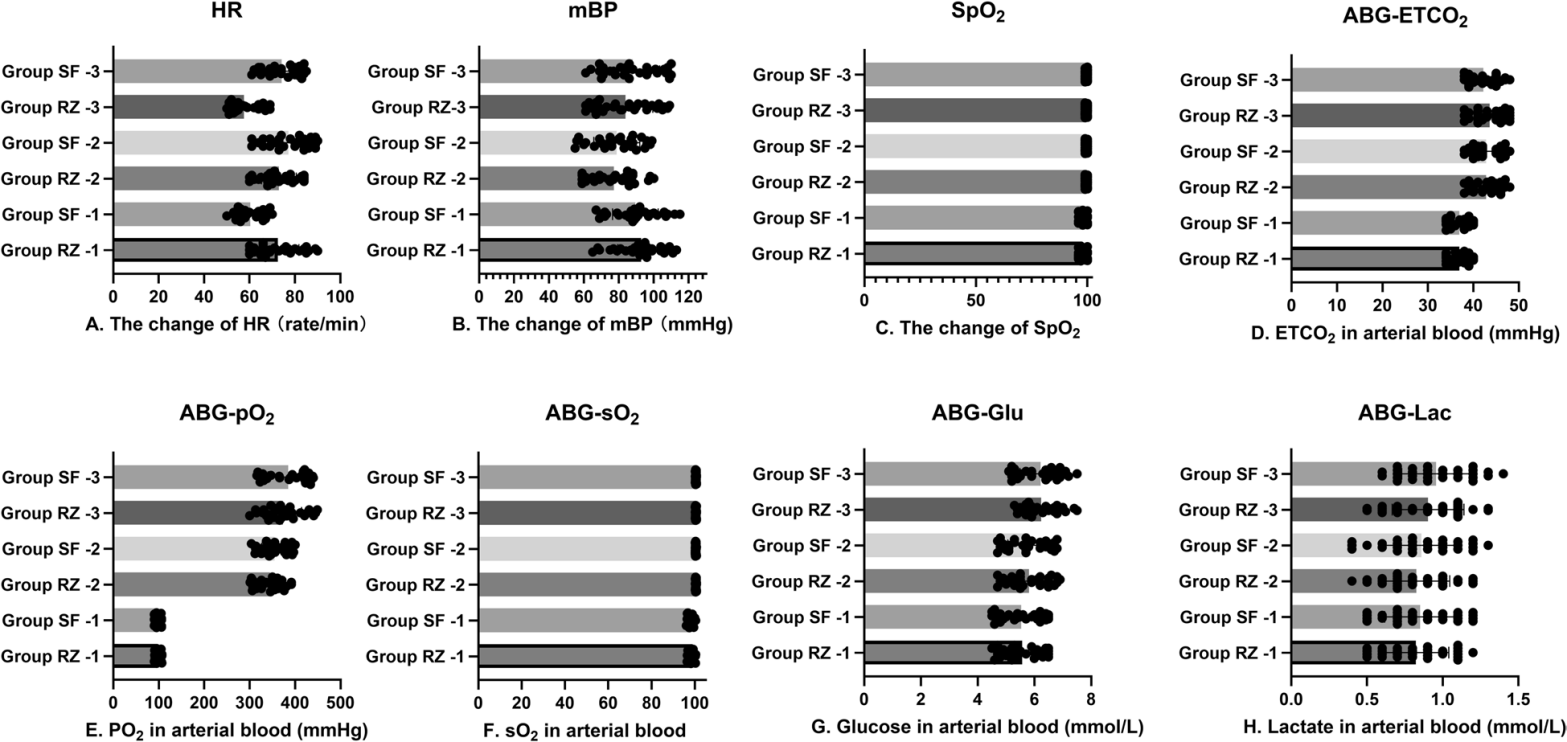
Comparison of vital signs and arterial blood gas (ABG) results between two groups of patients at three time points, such as **A** heart rate (HR), **B** mean blood pressure (mBP), **C** blood oxygen saturation (SpO_2_), **D** end of breath carbon dioxide (ETCO_2_), **E** arterial oxygen partial pressure (pO_2_), **F** blood gas oxygen saturation, **G** blood glucose (Glu) levels in ABG, and **H** blood lactate (Lac) levels in ABG. All the above indicators have no statistical significance in both groups. *P* < 0.05 was statistically significant

There were no statistically significant differences between the two groups in the changes in heart rate, blood pressure, and oxygen saturation recorded at the three time points, nor in the arterial blood gas analysis results obtained at these time points (Fig. 3).

**Fig. 4:**
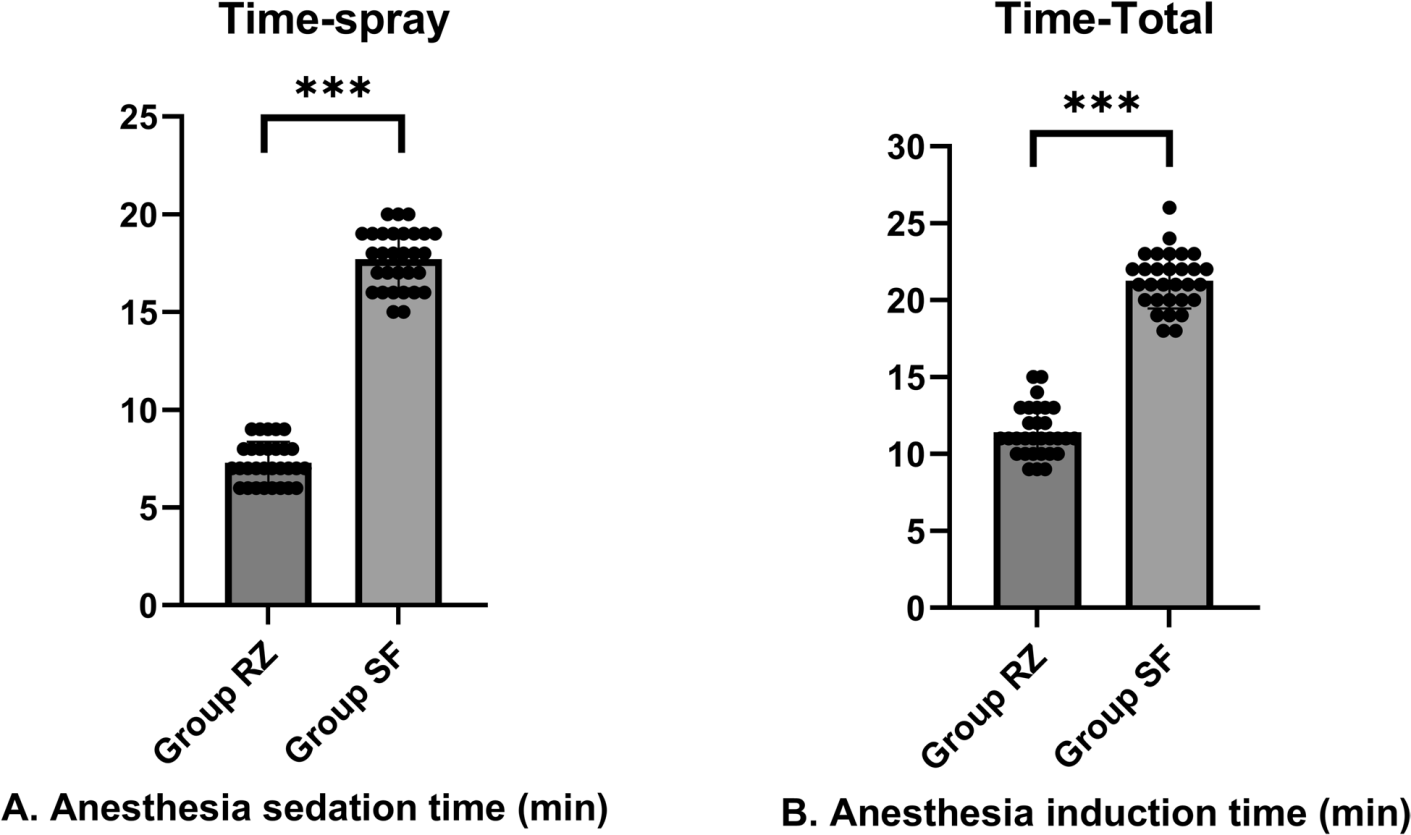
Comparison of anesthesia induction time between two groups of patients. **A** Compared with the two groups of patients, the RZ group had a significantly shorter time to reach the anesthesia depth that could be sprayed into the throat. Wilcoxon rank sum test: *P* value 0.000. 95% confidence interval, 9.733 to 11.07. **B** Compared with the two groups of patients, the RZ group had a significantly shorter time to reach the anesthesia depth that could allow for successful intubation. Wilcoxon rank sum test: *P* value 0.000. 95% confidence interval, 8.979 to 10.75. ***P < 0.01. *P* < 0.05 was statistically significant

Regarding the total time required for anesthetic induction, the remimazolam group achieved the conditions necessary for throat spraying in an average of 7.3 min, whereas the sevoflurane group required an average of 17.7 min, which was statistically significant. The total time to successful intubation was 11.4 min on average in the remimazolam group and 21.3 min in the sevoflurane group, which was statistically significant (Fig. 4).

## Discussion

The present work is the first to apply remimazolam tosilate for anesthesia during tracheal intubation while maintaining spontaneous respiration to evaluate its effectiveness and safety compared with sevoflurane. The key findings of this study are as follows: (1) remimazolam can be as safely applied as sevoflurane for anesthesia induction and intubation while preserving spontaneous respiration, both groups showed no severe complications, no hypoxia during induction, and successful glottic exposure, throat spray, and intubation; (2) during the process of achieving sufficient anesthetic depth, patients in the remimazolam group remained calm, whereas 37% of patients in the sevoflurane group exhibited body movement during the anesthesia excitement phase; (3) under the condition of mask nebulized local anesthetic, both groups successfully exposed the glottis with low stress responses, but coughing during throat spray was significantly higher in the remimazolam group than in the sevoflurane group. After successful throat spray, both groups showed a significant reduction in body movement and coughing during intubation, with no statistically significant difference between them; (4) both groups achieved sufficient anesthetic depth (as assessed by EEG and MOAA/S), but the remimazolam group took significantly shorter anesthesia sedation time and anesthesia induction time.

In clinical anesthesia, situations often arise where it is necessary to perform tracheal intubation while preserving spontaneous respiration, such as in patients with neck masses, obstructive sleep apnea, or known or potential difficult airways (Ahmad et al. 2020; Ahmad et al. 2019). While traditional awake intubation is safe, many patients cannot tolerate it, leading to severe coughing, vomiting, and significant hemodynamic fluctuations (Tomar 2016). Additionally, some patients may have contraindications for awake intubation, such as elderly patients with cardiovascular diseases, or those who are non-cooperative, such as the deaf, mute, or intellectually disabled (Aziz and Kristensen 2020; Wang et al. 2018; Li et al. 2015). In these cases, intubation under a certain level of sedation while maintaining spontaneous respiration is needed, but the challenge lies in successfully completing the intubation while minimizing stress responses and complications.

Sevoflurane, a short-acting inhalational anesthetic, is commonly used for tracheal intubation in patients with difficult airways. However, sevoflurane alone has an irritating odor that some patients find intolerable (Lee et al. 2024a; Wan et al. 2019). Remimazolam tosylate (RMB), a newly developed benzodiazepine currently undergoing clinical trials, is an ultra-short-acting intravenous sedative-hypnotic. It exerts its effects by acting on GABAA receptors, increasing chloride ion permeability in neuronal membranes, leading to hyperpolarization and inhibition of neuronal activity, resulting in sedation (Johnson et al. 2024). As an ultra-short-acting GABAA receptor agonist, remimazolam is metabolized by plasma esterases without relying on liver or kidney function (Sneyd 2022). Its metabolite, remimazolam carboxylic acid, has no pharmacological activity. Remimazolam has a rapid onset and offset of action, with short sedation recovery time, linear pharmacokinetics, a short half-life, and its clearance is independent of body weight (Katsuragawa et al. 2023; Shi, et al. 2024; Dai et al. 2021; Eisenried et al. 2020). Additionally, its sedative effects can be quickly reversed by flumazenil, making it safe and controllable (Barbosa et al. 2024). Compared to opioids and propofol, remimazolam has a milder effect on respiratory and cardiovascular depression (Lee et al. 2024a; Yoo et al. 2024). Given its pharmacokinetic advantages, these properties make remimazolam a promising option for situations requiring precise control over sedation depth and duration. Remimazolam may be particularly useful for short-duration procedures requiring sedation with spontaneous respiration, such as endoscopic interventions and bronchoscopy (Xu et al. 2024). Its predictable metabolism makes it a safer option for patients with hepatic or renal dysfunction.

Previous studies have demonstrated that patients sedated with remimazolam exhibit higher satisfaction compared to those receiving other sedatives, such as propofol or dexmedetomidine (Shi et al. 2022a). Our findings are consistent with these observations. During the induction phase, unlike the discomfort associated with the pungent odor of sevoflurane, none of the patients in the RZ group reported injection pain. This may be attributed to the water-soluble nature of remimazolam, which eliminates the need for emulsifying agents, thereby reducing vascular irritation. Also, remimazolam does not activate nociceptors or induce pain signaling, contributing to its superior tolerability (Shi et al. 2022b). Furthermore, in Line with previous research, patients in the remimazolam group remained calm throughout the process of achieving sufficient anesthetic depth and exhibited a significantly shorter induction time. In contrast, the sevoflurane group required a longer duration to achieve an adequate depth of anesthesia, and 37% of patients in the sevoflurane group demonstrated involuntary body movements during the anesthesia excitement phase. This finding suggests that additional physical restraint may be necessary in the sevoflurane group to prevent adverse events, such as accidental falls.

Topical anesthesia of the supraglottic, glottic, and subglottic regions prior to tracheal intubation significantly mitigates airway-related adverse reactions, including coughing and bronchospasm, and plays a critical role in maintaining spontaneous respiration during awake intubation (Li et al. 2016). However, under the condition of mask nebulized local anesthetic, both groups successfully exposed the glottis with low stress responses, although coughing during throat spray was significantly higher in the remimazolam group than in the sevoflurane group. This indicates that the effectiveness of mask nebulized local anesthetic in reaching below the glottis was less, but it was sufficient to anesthetize the supraglottic oropharyngeal area for optimal video laryngoscope conditions. Following successful throat spray administration, both groups exhibited a significant reduction in body movement and coughing during intubation, with no statistically significant difference observed between the two. The results of this study suggest that remimazolam can be as safely applied as sevoflurane for anesthesia induction and intubation while preserving spontaneous respiration. Future research should investigate the combination of remimazolam with adjuncts like opioids to optimize airway management while refining dosing regimens and administration strategies to enhance safety and patient comfort.

Moreover, in perioperative management, the rapid recovery of postoperative cognitive function is of vital importance. The impact of remimazolam, a novel benzodiazepine derivative, on postoperative cognitive function in patients, especially the elderly, has been a subject of controversy (Yang et al. 2021; Aoki et al. 2023; Kaneko et al. 2023). Both study groups in this trial received dexmedetomidine as part of their anesthetic regimen. Dexmedetomidine, a multifunctional agent with sedative, analgesic, anxiolytic, sympatholytic, and opioid-sparing properties, is particularly valued for its preservation of respiratory function. This characteristic enables its widespread application in scenarios requiring maintenance of spontaneous respiration. As a background medication for preserving spontaneous breathing, the inclusion of dexmedetomidine in both the control and experimental groups does not compromise the scientific validity of the remimazolam-sevoflurane comparison. This clinically relevant trial design not only enhances patient comfort but also strengthens the safety and efficacy of spontaneous respiration maintenance (Lee et al. 2024b; Gao et al. 2023).

However, the limitations of this study include the fact that all enrolled patients were selected after airway assessment, excluding those with potential ventilation difficulties, intubation challenges, and obesity. Whether remimazolam can still achieve sufficient safety and effectiveness during anesthesia induction with preserved spontaneous breathing in these patients requires further research. Additionally, the study population excluded pediatric and elderly patients. Whether remimazolam can be safely used in these populations also necessitates further investigation by more researchers.

## Conclusion

Our study confirms that remimazolam provides a safe and effective alternative to sevoflurane for tracheal intubation while preserving spontaneous respiration. Its rapid onset, short duration of action, minimal impact on hemodynamics, and availability of a reversal agent make it a valuable addition to anesthetic practice. Future research should focus on optimizing dosing strategies to minimize airway irritation while maintaining effective sedation in patients with difficult airways.

## Data Availability

All the data supporting our findings are contained within the manuscript.

## References

[CR1] Ahmad I, et al. Airway management research: a systematic review. Anaesthesia. 2019;74(2):225–36. 30460982 10.1111/anae.14471

[CR2] Ahmad I, et al. Difficult Airway Society guidelines for awake tracheal intubation (ATI) in adults. Anaesthesia. 2020;75(4):509–28. 31729018 10.1111/anae.14904 PMC7078877

[CR3] Aoki Y, et al. Association between remimazolam and postoperative delirium in older adults undergoing elective cardiovascular surgery: a prospective cohort study. J Anesth. 2023;37(1):13–22. 36220948 10.1007/s00540-022-03119-7

[CR4] Aziz MF, Kristensen MS. From variance to guidance for awake tracheal intubation. Anaesthesia. 2020;75(4):442–6. 31828761 10.1111/anae.14947

[CR5] Barbosa EC, et al. Remimazolam versus propofol for sedation in gastrointestinal endoscopic procedures: a systematic review and meta-analysis. Br J Anaesth. 2024;132(6):1219–29. 38443286 10.1016/j.bja.2024.02.005

[CR6] Caraballo C, et al. Clinical implications of the New York Heart Association classification. J Am Heart Assoc. 2019;8(23): e014240. 31771438 10.1161/JAHA.119.014240 PMC6912957

[CR7] Chernik DA, et al. Validity and reliability of the Observer’s Assessment of Alertness/Sedation Scale: study with intravenous midazolam. J Clin Psychopharmacol. 1990;10(4):244–51. 2286697

[CR8] Dai G, et al. Safety and efficacy of remimazolam compared with propofol in induction of general anesthesia. Minerva Anestesiol. 2021;87(10):1073–9. 34263581 10.23736/S0375-9393.21.15517-8

[CR9] Eisenried A, et al. Pharmacokinetics and pharmacodynamics of remimazolam (cns 7056) after continuous infusion in healthy male volunteers: part II. Pharmacodynamics of Electroencephalogram Effects Anesthesiology. 2020;132(4):652–66. 31972657 10.1097/ALN.0000000000003102

[CR10] Gao S, et al. Clinical effects of remimazolam alone or in combination with dexmedetomidine in patients receiving bronchoscopy and influences on postoperative cognitive function: a randomized-controlled trial. Int J Clin Pharm. 2023;45(1):137–45. 36346544 10.1007/s11096-022-01487-4

[CR11] Hu Q, et al. Remimazolam: an updated review of a new sedative and anaesthetic. Drug des Devel Ther. 2022;16:3957–74. 36411859 10.2147/DDDT.S384155 PMC9675580

[CR12] Johnson KB. New horizons in sedative hypnotic drug development: fast, clean, and soft. Anesth Analg. 2012;115(2):220–2. 22826519 10.1213/ANE.0b013e31825ef8d7

[CR13] Johnson KL, et al. Remimazolam: a retrospective study of initial safety and recovery data in diverse procedural sedation. Clin Ther. 2024;46(2):90–5. 38071132 10.1016/j.clinthera.2023.11.004

[CR14] Kaneko S, et al. Effect of remimazolam on the incidence of delirium after transcatheter aortic valve implantation under general anesthesia: a retrospective exploratory study. J Anesth. 2023;37(2):210–8. 36463532 10.1007/s00540-022-03148-2

[CR15] Katsuragawa T, et al. Effect of remimazolam versus sevoflurane on intraoperative hemodynamics in noncardiac surgery: a retrospective observational study using propensity score matching. JA Clin Rep. 2023;9(1):70. 37880547 10.1186/s40981-023-00661-5 PMC10600086

[CR16] Lee J, et al. Comparison of Remimazolam versus Sevoflurane on the postoperative quality of recovery in cervical spine surgery: a prospective randomized controlled double-blind trial. Drug des Devel Ther. 2024a;18:121–32. 38283136 10.2147/DDDT.S441622 PMC10821644

[CR17] Lee S, et al. Retrospective comparison of the effects of remimazolam and dexmedetomidine on postoperative delirium in elderly patients undergoing orthopedic surgery of the lower extremities under spinal anesthesia. J Anesth. 2024b;38(6):771–9. 39182205 10.1007/s00540-024-03386-6

[CR18] Li P, et al. Assessment of tracheal intubation in adults after induction with sevoflurane and different doses of propofol: a randomly controlled trial. Int J Clin Exp Med. 2015;8(8):14042–7. 26550365 PMC4613050

[CR19] Li LW, et al. Site-directed topical lidocaine spray attenuates perioperative respiratory adverse events in children undergoing elective surgery. J Surg Res. 2016;203(1):206–10. 27338551 10.1016/j.jss.2016.03.011

[CR20] McEvoy JW, et al. 2024 ESC Guidelines for the management of elevated blood pressure and hypertension. Eur Heart J. 2024;45(38):3912–4018. 39210715 10.1093/eurheartj/ehae178

[CR21] Shi F, et al. Application of remimazolam-0.6% sevoflurane anesthesia for flash visual evoked potential monitoring during pituitary adenoma resection: a non-inferiority randomized controlled trial. BMC Anesthesiol. 2024;24(1):85. 38424486 10.1186/s12871-024-02466-0 PMC10903035

[CR22] Shi F, et al. Efficacy and safety of remimazolam tosilate versus propofol for general anesthesia in cirrhotic patients undergoing endoscopic variceal ligation. Int J Gen Med. 2022a;15:583–91. 35046716 10.2147/IJGM.S345390 PMC8763269

[CR23] Shi W, et al. Efficacy and safety of the remimazolam-alfentanil combination for sedation during gastroscopy: a randomized, double-blind, single-center controlled trial. Clin Ther. 2022b;44(11):1506–18. 36763995 10.1016/j.clinthera.2022.09.014

[CR24] Sneyd JR. Remimazolam: new beginnings or just a me-too? Anesth Analg. 2012;115(2):217–9. 22826518 10.1213/ANE.0b013e31823acb95

[CR25] Sneyd JR. Avoiding kidney damage in ICU sedation with sevoflurane: use isoflurane instead. Br J Anaesth. 2022;129(1):7–10. 35331541 10.1016/j.bja.2022.02.031

[CR26] Tomar GS. Difficult airway society 2015 guidelines for management of unanticipated difficult intubation in adults: need to be revisited? Br J Anaesth. 2016;117(4):529. 28077542 10.1093/bja/aew278

[CR27] Wan L, et al. Dexmedetomidine reduces sevoflurane EC50 for supraglottic airway device insertion in spontaneously breathing morbidly obese patients. Ther Clin Risk Manag. 2019;15:627–35. 31118650 10.2147/TCRM.S199440 PMC6504637

[CR28] Wang J, et al. Effectiveness and safety of a novel approach for management of patients with potential difficult mask ventilation and tracheal intubation: a multi-center randomized trial. Chin Med J. 2018;131(6):631–7. 29521283 10.4103/0366-6999.226897 PMC5865306

[CR29] Wang Z, et al. Fiberoptic-guided tracheal intubation under precise anesthesia and topicalization with spontaneous respiration preservation for an uncooperative patient with severe postburn mentosternal contracture. Clin Case Rep. 2021;9(12): e05208. 34934504 10.1002/ccr3.5208 PMC8650747

[CR30] Wesolowski AM, et al. Remimazolam: pharmacologic considerations and clinical role in anesthesiology. Pharmacotherapy. 2016;36(9):1021–7. 27496519 10.1002/phar.1806

[CR31] Xu H, et al. Comparison of the safety and efficacy of remimazolam besylate versus dexmedetomidine for patients undergoing fiberoptic bronchoscopy: a prospective, randomized controlled trial. Drug des Devel Ther. 2024;18:2317–27. 38915861 10.2147/DDDT.S460949 PMC11194170

[CR32] Yang M, et al. Effect of remimazolam besylate compared with propofol on the incidence of delirium after cardiac surgery: study protocol for a randomized trial. Trials. 2021;22(1):717. 34663423 10.1186/s13063-021-05691-x PMC8522864

[CR33] Yoo YM, et al. The incidences of nausea and vomiting after general anesthesia with remimazolam versus sevoflurane: a prospective randomized controlled trial. Korean J Anesthesiol. 2024;77(4):441–9. 38637272 10.4097/kja.23939 PMC11294881

[CR34] Zhou Y, et al. Spontaneous breathing anesthesia for cervical tracheal resection and reconstruction. J Thorac Dis. 2019;11(12):5336–42. 32030251 10.21037/jtd.2019.11.70 PMC6988036

